# Recurrence rate and postoperative fistula formation: A retrospective analysis of surgically managed cases of anorectal abscess

**DOI:** 10.5339/qmj.2025.101

**Published:** 2025-12-02

**Authors:** Saleh A. Busbait

**Affiliations:** 1 Department of Surgery, College of Medicine, Imam Abdulrahman Bin Faisal University, Dammam, Saudi Arabia *Email: sbusbait@iau.edu.sa

**Keywords:** Abscess, fistula, recurrence, drainage, risk factors, treatment outcomes

## Abstract

**Background:**

Anorectal abscesses are common surgical conditions associated with a high risk of recurrence or fistula formation. Although several studies have investigated the potential predictors, data from Saudi Arabia remain limited. This study aimed to assess the rate, timing, and predictors of recurrence or fistula formation following the primary surgical drainage of anorectal abscesses.

**Methods:**

We conducted a five-year retrospective cohort study at King Fahad Hospital of the University in Al Khobar, Saudi Arabia, including all patients who underwent surgical incision and drainage for a first-time anorectal abscess between January 2019 and December 2023. Demographic data, abscess characteristics, operative details, and follow-up outcomes were analyzed. Logistic regression and Cox proportional hazards models were used to identify the predictive factors.

**Results:**

Among 302 patients, 51.7% (*n* = 156) developed either a recurrent abscess (12.9%, *n* = 39) or an index perianal fistula (38.7%, *n* = 117) during follow-up. The mean time to recurrence was 19.5 weeks. Complex abscesses, observed in 28.1% (*n* = 85) of cases, were the only independent predictor of earlier recurrence (hazard ratios [HR]: 2.391, *p* < 0.001). Preoperative imaging was also associated with an increased risk in logistic regression analysis. Seton placement was rare (2.6%, *n* = 8), despite intraoperative fistula detection in 17.9% (*n* = 54) of cases.

**Conclusion:**

Recurrence and fistula formation following drainage of first-time anorectal abscesses are common and tend to occur early. Abscess complexity is a key predictor of poor outcomes, highlighting the need for structured early follow-up and risk-based surgical planning.

## 1. INTRODUCTION

Anorectal abscess is a common surgical condition, accounting for a significant portion of emergency presentations in both outpatient and inpatient settings.^1,2^ It predominantly affects young adult males and typically arises from cryptoglandular infections originating in the anal glands.^1^ In a large population-based study, males represented approximately 70% of affected patients.^3^ Surgical incision and drainage are the initial treatment for most patients; however, a significant proportion subsequently develop recurrent abscesses or perianal fistulas.^4,5^

In the published literature, the occurrence or recurrence of a fistula-in-ano ranges from 17% to 50% of cases. Reported rates vary depending on the follow-up duration, case definitions used, and whether intraoperative fistula detection is included.^1,3–7^ In most cases, fistulas develop within the first 3–6 months after abscess drainage.^5^ However, predicting which patients are likely to progress to chronic fistulizing disease remains challenging. Studies have examined risk factors—including abscess complexity, comorbidities such as diabetes, and the use of preoperative imaging—with inconsistent findings.^2,4^

Although anorectal abscess is highly prevalent in the region, there is a lack of studies from Saudi Arabia examining recurrence rates, predictive factors, and practice patterns related to intraoperative findings or seton use. Given these limitations, this study aims to evaluate the outcomes of patients presenting for the first time with an anorectal abscess at a tertiary academic center. Additionally, we assess the factors associated with recurrence or fistula formation. The findings of this study are intended to support the development of early follow-up strategies and to inform future improvements in surgical decision-making and outpatient management.

## 2. MATERIALS AND METHODS

This retrospective cohort study was conducted at King Fahad Hospital of the University in Al Khobar, a tertiary academic medical center in Saudi Arabia.

### 2.1. Inclusion criteria

The study included all patients who underwent surgical incision and drainage for a first-time anorectal abscess between January 1, 2019, and December 31, 2023. Patient selection was performed using International Classification of Diseases (ICD) codes and operative notes. All cases were manually reviewed to confirm eligibility and minimize misclassification. All surgical drainage procedures were performed in the operating room under sterile conditions.

### 2.2. Exclusion criteria

Patients were excluded if they were younger than 13 years at initial presentation, had a prior history of recurrent abscess or chronic perianal fistula, or had non-cryptoglandular causes of abscess such as Crohn’s disease, malignancy, tuberculosis, obstetric trauma, prior rectal surgery, immunosuppressive conditions, or foreign body-associated abscesses. Patients who developed recurrence within 10 days and required early revision surgery due to suspected inadequate drainage were also excluded.

A non-probability consecutive sampling method was employed, including all eligible patients who met the inclusion criteria during the study period. Clinical data were retrieved from the hospital’s Health Information System (HIS) electronic medical records and subsequently exported to IBM SPSS Statistics version 30.0 for analysis. Collected variables included age at presentation, gender, smoking status, body mass index (BMI), presence of diabetes mellitus, hemoglobin A1c (HbA1c) level (when available), white blood cell (WBC) count, C-reactive protein (CRP), abscess location and complexity, number of abscesses, intraoperative fistula detection, preoperative imaging modality (computed tomography [CT], magnetic resonance imaging [MRI], or ultrasound), perioperative antibiotic use and duration, and operating surgeon’s level (resident, specialist, or consultant).

### 2.3. Operational definitions

Abscesses were classified intraoperatively as either simple or complex based on predefined clinical criteria. The abscesses were considered complex if they formed multiple interconnected cavities, were associated with fistula-in-ano identified during surgery, or demonstrated tissue spread beyond the perianal space (including ischiorectal, intersphincteric, or supralevator areas). All other abscesses were classified as simple. Incision and drainage procedures were performed by multiple surgeons, reflecting real-world clinical practice in a tertiary care center. Procedures were performed in the operating room under sterile conditions, with the patient positioned in lithotomy. A standardized surgical technique was applied in all cases, including stab incision, opening of all loculi, irrigation with normal saline and diluted povidone-iodine, and gauze packing. Adjunctive measures, such as seton placement or additional incisions in cases with multiple abscesses, were performed at the discretion of the operating surgeon.

Intraoperative identification of fistulas and placement of setons were documented. Preoperative antibiotics were documented for cases in which the medication was administered within 24 hours prior to surgery, while postoperative antibiotics were documented when prescribed at discharge, along with the duration of treatment in days. All categorical variables were numerically coded and appropriately labeled for analysis. Missing values were handled using system-missing codes in SPSS.

The composite primary outcome comprised both the clinical recurrence of anorectal abscess and the development of an index fistula following the initial surgical drainage. Fistulas identified either intraoperatively or during follow-up were counted as events. The intraoperative and delayed fistula diagnoses were recorded separately but analyzed together as part of the composite recurrence/fistula outcome. Recurrence was defined as the need for repeat surgical drainage due to a new abscess forming in the same or adjacent perianal area at any point during follow-up. A definitive diagnosis of fistula during follow-up was established based on physical examination and/or pelvic MRI when available. A clinical diagnosis was made if persistent perianal suppuration or an external opening was observed more than eight weeks after the initial drainage. Follow-up duration was calculated from the date of initial surgery until either the date of documented recurrence/fistula or the last recorded surgical clinic visit. Duration was recorded in weeks for descriptive analyses and in days or weeks for time-to-event analyses, as appropriate.

Descriptive statistics were used to summarize the baseline characteristics. Categorical variables were compared using chi-square tests. Two regression models were used to identify predictors of recurrence or fistula: (1) binary logistic regression for the binary outcome of recurrence/fistula and (2) Cox proportional hazards regression to evaluate time to event. Hazard ratios (HR), odds ratios (OR), and 95% confidence intervals (CI) were reported. Variables with a p-value <0.25 in the univariable Cox analysis or considered clinically relevant were included in the multivariable model. Statistical significance was defined as a p-value <0.05 or a 95% CI that did not include 1.0.

The study was approved by the Institutional Review Board (IRB) and Ethics Committee at Imam Abdulrahman Bin Faisal University (IRB number: IRB-2024-01-233), with approval granted on March 11, 2024. The study posed no physical, psychological, legal, or economic risk to participants and adhered to the ethical research guidelines established by the IRB. Individual informed consent was not required, as the data were collected anonymously.

## 3. RESULTS

A total of 302 patients with perianal abscess were included in the study. Baseline demographic and clinical characteristics are summarized in Table 1. The mean age was 37.9 ± 13.5 years, with 60.9% (*n* = 184) of patients younger than 40 years. Males constituted the majority of the cohort (75.8%, *n* = 229). The mean BMI was 27.6 ± 6.6 kg/m^2^, indicating that 57.9% (*n* = 175) of the cohort were overweight or obese (BMI ≥ 25). The prevalence of smoking and diabetes mellitus was 4.6% (*n* = 14) and 27.8% (*n* = 84), respectively.

Inflammatory markers were available for a subset of patients. CRP levels were recorded for 222 patients, with a mean of 9.5 ± 9.3 mg/dL; 96.4% (*n* = 214) had CRP levels exceeding 0.1 mg/dL. WBC counts were available for 299 patients, with a mean of 12.6 ± 5.9×10^9^/L; 55.5% (*n* = 166) had abnormal WBC levels (<4 or >11 ×10^9^/L). HbA1c was measured in 81 patients (those with diabetes or high-risk features), with a mean of 9.95 ± 2.77%; among them, 90.1% (*n* = 73) had poorly controlled glycemia (HbA1c ≥ 6.5%). The mean follow-up duration was 30.8 ± 44.5 weeks. The mean time to development of fistula or abscess recurrence among the 156 patients with these outcomes was 19.5 ± 29.8 weeks. Of these 156 patients, 117 (38.7%) developed a perianal fistula, while 39 (12.9%) developed a new abscess.

As shown in Table 2, the most common abscess site was perianal (82.5%, *n* = 249), followed by ischiorectal (14.2%, *n* = 43), intersphincteric (2.0%, *n* = 6), and supralevator (1.0%, *n* = 3). Abscesses were classified as simple in 71.9% (*n* = 217) of cases and complex in 28.1% (*n* = 85). A single abscess was observed in 92.4% (*n* = 279) of patients, while 7.6% (*n* = 23) had multiple abscesses. Intraoperative fistula detection occurred in 17.9% (*n* = 54) of patients. Most fistulas were identified by specialists (37.0%, *n* = 20), followed by consultants (25.9%, *n* = 14), and residents (1.9%, *n* = 1). Seton placement was performed in only 2.6% (*n* = 8) of patients, all operated on by consultants. Notably, 57.1% (*n* = 8) of the fistulas detected by consultants were managed with a seton, indicating that only 14.8% (*n* = 8) of all patients with an intraoperatively detected fistula underwent seton placement.

Preoperative imaging was performed in 13.6% (*n* = 41) of patients—most commonly with CT (10.9%, *n* = 33), followed by MRI (2.0%, *n* = 6) and ultrasound (0.7%, *n* = 2). The majority of patients (86.4%, *n* = 261) underwent surgery without any preoperative imaging. All patients received preoperative antibiotics, and 92.4% (*n* = 279) received postoperative antibiotics. Preoperative antibiotics were administered empirically according to surgeon preference, with the most common regimens being ciprofloxacin plus metronidazole (45.0%), Augmentin plus metronidazole (14.9%), or Augmentin alone (11.6%). Among those who received postoperative antibiotics (*n* = 276), the majority (67.4%, *n* = 186) were treated for fewer than seven days, while 27.5% (*n* = 76) received treatment for 7–14 days, and 5.1% (*n* = 14) received antibiotics for more than 14 days. The operating surgeon was a specialist in 52.6% of cases (*n* = 159), a consultant in 43.4%(*n* = 131), and a resident in 4.0% (*n* = 12).

The results from the univariable analysis identified several factors associated with recurrence or fistula formation (Table 3). The recurrence or fistula formation rate was significantly higher in patients aged 40 years or older (59.3%, *n* = 70) than in those younger than 40 years (46.7%, *n* = 86; *p* = 0.033). There was no significant difference in recurrence based on gender (male: 51.5%, *n* = 118 vs. female: 52.1%, *n* = 38; *p* = 0.938), BMI category (BMI < 25: 48.8%, *n* = 62 vs. BMI ≥ 25: 53.7%, *n* = 94; *p* = 0.401), diabetes status (diabetic: 46.4%, n=39 vs. non-diabetic: 53.7%, *n* = 117; *p* = 0.259), or WBC level (≤11: 48.1%, *n* = 64 vs. >11: 53.6%, *n* = 89; *p* = 0.345).

The recurrence rate was lower among smokers than non-smokers (21.4%, *n* = 3 vs. 53.1%, *n* = 153; *p* = 0.020), whereas elevated CRP levels (>5 mg/L) were not significantly associated with recurrence (51.2%, *n* = 65 vs. 40.4%, *n* = 44; *p* = 0.440). Among patients with recorded HbA1c, those with poor glycemic control (≥ 6.5%) had a lower recurrence rate than those with better control (43.8%, *n* = 32 vs. 75.0%, *n* = 6; *p* = 0.094); although this difference was not statistically significant.

Analysis of abscess characteristics showed that complex abscesses had a higher recurrence rate than simple abscesses (75.3%, *n* = 64 vs. 42.4%, *n* = 92; *p* < 0.001). The number of abscesses was not significantly associated with recurrence rates (multiple: 60.9%, *n* = 14 vs. single: 50.9%, *n* = 142; *p* = 0.358), although patients who had multiple abscesses showed higher numerical recurrence rates. The recurrence rate was higher among patients with supralevator and intersphincteric abscesses compared with those with perianal and ischiorectal types; however, this result was not statistically significant (*p* = 0.194).

Patients who underwent preoperative imaging had a higher recurrence rate than those who did not (68.3%, *n* = 28 vs. 49.0%, *n* = 128; *p* = 0.022). Recurrence rates varied by operating surgeon’s level (consultant: 54.2%, *n* = 71; specialist: 48.4%, *n* = 77; resident: 66.7%, *n* = 8; *p* = 0.353) and by postoperative antibiotic duration (49.5%–71.4%, *p* = 0.452); although these differences were not statistically significant.

A multivariable binary logistic regression analysis was performed to identify independent predictors of recurrence or fistula formation following perianal abscess drainage. The final model included age, gender, BMI, diabetes status, WBC count, CRP level, preoperative imaging, and postoperative antibiotic duration as covariates (Table 4). Among the included predictors, higher CRP levels (OR: 0.950, 95% CI: 0.908–0.994, *p* = 0.028) were significantly associated with lower odds of recurrence or fistula formation, indicating a potential inverse relationship. Additionally, preoperative imaging was significantly associated with higher odds of recurrence/fistula formation (OR: 2.987, 95% CI: 1.208–7.383, *p* = 0.018).

The WBC count showed a borderline association with recurrence or fistula formation (OR: 1.067, 95% CI: 0.999–1.140, *p* = 0.052), suggesting a possible trend toward increased risk with higher WBC levels. The odds of recurrence or fistula formation were lower in diabetic patients (OR: 0.484, 95% CI: 0.232–1.008), though this finding was not statistically significant (*p* = 0.052). Other factors, including age, gender, BMI, and postoperative antibiotic duration (treated as a continuous variable), were not significantly associated with the outcome.

Table 5 presents the results of the univariable and multivariable Cox proportional hazards regression analysis for time to abscess recurrence or fistula formation. The univariable analysis indicated that abscess complexity was a significant predictor of increased hazard, with patients having complex abscesses exhibiting more than twice the hazard of those with simple abscesses (HR: 2.260, 95% CI: 1.640–3.110, *p* < 0.001). Pre-drainage imaging showed a trend toward significance (HR: 1.470, 95% CI: 0.980–2.220, *p* = 0.065), whereas age, gender, smoking, and diabetes were not statistically significant predictors (*p* > 0.05 for all).

The multivariable analysis showed that abscess complexity was the only factor significantly predicting recurrence or fistula formation, with an HR of 2.391 (95% CI: 1.658–3.450, *p* < 0.001). The adjusted model showed that age (HR: 1.000, *p* = 0.978), gender (HR: 0.954, *p* = 0.806), smoking (HR: 0.564, *p* = 0.333), diabetes (HR: 0.836, *p* = 0.390), and preoperative imaging (HR: 0.936, *p* = 0.780) were not significant predictors.

## 4. DISCUSSION

We evaluated 302 patients who underwent incision and drainage for a first-time anorectal abscess at a tertiary academic center, assessing recurrence and fistula formation over five years. Overall, 51.7% (*n* = 156) of patients developed either a recurrent abscess or an index perianal fistula during follow-up. The recurrent anorectal abscesses occurred in 12.9% (*n* = 39) of patients, while perianal fistulas developed in 38.7% (*n* = 117). Most of these events occurred early, with a mean time to recurrence or fistula formation of 19.5 ± 29.8 weeks and a mean follow-up duration of 30.8 ± 44.5 weeks. Fistulas identified intraoperatively were included in the composite outcome because of their clinical relevance to recurrence risk.

The study population was predominantly male (75.8%, *n* = 229), with most patients younger than 40 years (60.9%, *n* = 184) and overweight or obese (57.9%, *n* = 175). The most common abscess site was perianal (82.5%, *n* = 249), followed by ischiorectal (14.2%, *n* = 43), with complex abscesses observed in 28.1% (*n* = 85) of cases.

Intraoperative fistulas were detected in 17.9% of cases (*n* = 54), but seton placement was performed in only 2.6% (*n* = 8), representing 15% of the intraoperative fistulas identified. All setons were placed by consultant-level surgeons. Postoperative antibiotics were prescribed to 92.4% (*n* = 279) of patients, with 67.4% (*n* = 186) receiving treatment for fewer than seven days.

The binary logistic regression model (Table 4) identified preoperative imaging and CRP levels as significant predictors. Patients who underwent preoperative imaging had nearly three-fold higher odds of recurrence or fistula formation (OR: 2.987, 95% CI: 1.208–7.383, *p* = 0.018). The results likely reflect selection bias, as imaging was more frequently performed in patients with atypical presentations or suspected complex disease, rather than serving as an independent risk factor. The findings of this study are consistent with those of Hamadani et al. and Yano et al., who reported that preoperative imaging was more frequently performed in complex or recurrent cases.^8,9^

Higher CRP levels were inversely associated with recurrence or fistula formation (OR: 0.950, 95% CI: 0.908–0.994, *p* = 0.028). This contrasts with previous studies, including a recent multicenter analysis in which CRP levels >100 mg/L were linked to significantly increased odds of persistent fistula following drainage (OR: 3.21, 95% CI: 1.57–6.71, *p* = 0.002).^10^ Although these results suggest that systemic inflammation may predict worse outcomes, our findings could instead reflect differences in presentation timing, patient selection, or the early use of targeted antibiotic in patients with higher CRP levels. Further prospective studies are needed to investigate this discrepancy.

Although diabetes and WBC count did not reach statistical significance in the final model, both showed borderline associations (*p* = 0.052). The inverse association observed with WBC count was unexpected, as elevated WBC count is typically linked to more severe infections. This finding may reflect earlier intervention or more aggressive initial treatment in patients presenting with higher WBC levels. Similar to CRP, further investigation in larger prospective cohorts is warranted to clarify the prognostic value of WBC in anorectal sepsis outcomes. Diabetes was associated with a lower odd of recurrence. Diabetic patients may have received more intensive follow-up and prolonged antibiotic therapy, potentially reducing their risk. A similar protective trend has been reported by Sánchez-Haro et al. and Sahnan et al.^3,4^ However, it is important to note that most prior studies, to our knowledge, assessed diabetes as a binary variable and did not stratify patients by glycemic control. The data remain mixed, and our observation of lower recurrence in patients with HbA1c ≥ 6.5% may reflect closer monitoring and more intensive management of poorly controlled diabetes, as has been similarly suggested in previous reports.^2,4,9^

The multivariable Cox proportional hazards model (Table 5) identified abscess complexity as the only significant predictor of earlier recurrence or fistula formation. Patients with complex abscesses had a 2.39-fold higher hazard of early recurrence (HR: 2.391, 95% CI: 1.658–3.450, *p* < 0.001). Demographic factors, including age, gender, BMI, and smoking status, were not significant predictors in the model.

The unadjusted chi-square analysis showed that patients older than 40 years experienced higher rates of recurrence or fistula formation (59.3%, *n* = 70 vs. 46.7%, *n* = 86; *p* = 0.033); however, this association was not significant in the adjusted model. Smoking appeared to be associated with lower recurrence rates (21.4%, *n* = 3 vs. 53.1%, *n* = 153; *p* = 0.020); however, this is likely due to underreporting, as only 14 patients (4.6%) were documented as smokers. The reported prevalence of smoking among adults in Saudi Arabia, particularly males, ranges from 15% to 21%, which is higher than observed in this study. This discrepancy suggests that smoking may be underreported in clinical records.^11^

The rates observed in our study are comparable to those reported in a recent large population-based study, which found that 70% of patients with anorectal abscesses were male and 20% developed a fistula during follow-up.^3^

Our cohort’s recurrence and fistula formation rate of 51.7% (*n* = 156) falls within the wide range reported in the literature, which varies from 17% to over 50% depending on study design, follow-up duration, and outcome definitions.^1,5,12^ The fistula formation rate of 38.7% (*n* = 117) aligns with the rates reported in regional studies, such as those of Hamadani et al. from Bahrain (26.5%) and Yano et al. from the U.S. (28%).^1,8,9^ Lower rates in Western cohorts may reflect differences in patient selection, shorter follow-up, or stricter exclusion of intraoperative fistulas.

Our study results show recurrence patterns consistent with previous research. We observed a median time to recurrence or fistula of six weeks, aligning with several studies reporting that most events occur within the first 3–6 months.^1,5,7^ Studies by Díaz et al. and Hasan et al. reported that late recurrences were infrequent, highlighting the importance of close monitoring following drainage procedures.^5,7^ Several regional studies reported outcomes based on short-term follow-up, usually not exceeding six months, potentially underestimating delayed recurrence and fistula formation rates.

The study results indicate that abscess complexity is the most significant predictor of adverse outcomes, aligning with previous research. Dong et al. also found that complex abscesses and less extensive surgical approaches were associated with higher recurrence rates.^13^ The risk of recurrence or fistula formation is significantly higher when abscesses are located in the ischiorectal, intersphincteric, or supralevator spaces.^4^

The logistic model showed a significant association between preoperative imaging and recurrence; however, this likely reflects selection bias, as observed in studies by Hamadani et al. and Yano et al., where imaging was more frequently performed for atypical or high-risk cases.^8,9^ While previous studies, such as that of Lu et al., found that elevated BMI was an independent risk factor for recurrence or fistula formation after anorectal abscess surgery (HR = 1.75, 95% CI: 1.15–2.67 for high BMI group), we did not observe a significant relationship in our cohort.^14^ This discrepancy may be attributable to differences in study populations, BMI distribution, or sample sizes.

Our demographic profile—predominantly male and below 40 years—is consistent with studies from Saudi Arabia, Bahrain, and other regions of Asia.^1,2^ Seton placement was infrequent (2.6%, *n* = 8), reflecting regional trends, despite a moderate rate of fistula detection during surgery.^7^ All seton placements in our study were performed by consultant surgeons, suggesting that the decision to place a seton was guided by the surgeon’s clinical judgment and experience. This contrasts with some prior reports in which most cases were managed by junior surgeons, suggesting that surgeon seniority may influence both intraoperative fistula detection and management decisions.^4^

Taken together, our findings support and extend previous reports, adding institution-specific data from a Saudi tertiary care center with structured follow-up and stratified surgical practice.

The high rates of recurrence and fistula formation observed in this study highlight the need for structured follow-up protocols, especially within the first 2–3 months post-drainage, when the majority of adverse outcomes occur. The findings indicate that early outpatient review should be prioritized for patients with complex abscesses, given their higher risk of recurrence, and for those undergoing preoperative imaging, which might indicate greater clinical severity.

The documentation of abscess complexity, fistula suspicion, and the operating surgeon’s level during routine intraoperative procedures should be emphasized, as these factors aid in risk stratification and follow-up planning. Although seton placement was rare, all cases were managed by consultants, suggesting that surgical experience may influence both the recognition and management of occult fistulas. Formalizing criteria for seton use and encouraging structured surgical assessment may improve clinical outcomes.

## 5. STRENGTHS AND LIMITATIONS

This study has several notable strengths. It presents one of the largest single-center cohorts in the region, focusing on first-time anorectal abscesses with comprehensive surgical records and follow-up data. Case verification was performed through manual review of operative notes and clinical records, minimizing the risk of misclassification. Compared to previous research, the study offers a more thorough evaluation of disease progression by assessing both intraoperative fistula detection and postoperative recurrence. Additionally, a median follow-up duration of over seven months enabled the detection of both early and delayed events, allowing for robust time-to-event analysis.

However, several limitations should be acknowledged. As a retrospective study, there is potential for selection bias and incomplete documentation, particularly for variables such as smoking. The involvement of multiple surgeons may have introduced variability in surgical techniques and decision-making, potentially affecting outcomes. The follow-up relied solely on the surgical clinic records, and the relatively short duration may have limited the detection of late recurrences, particularly in patients who sought care elsewhere. Decisions regarding imaging or seton placement were not standardized and depended on surgeon preference, which could introduce treatment bias. Finally, although intraoperative findings were recorded, variability in documentation quality across surgeon levels may have influenced fistula detection rates.

## 6. RECOMMENDATIONS

Future research should aim to validate these results through prospective studies with proper follow-up documentation. Evaluating structured imaging and seton placement protocols for clinical utility will help guide early intervention strategies and support consistent surgical decision-making. Future research involving prospective studies along with microbiological correlation should be conducted to develop improved predictive tools and management strategies. This area remains underexplored and will be addressed in a separate manuscript.

## 7. CONCLUSION

This retrospective cohort study of patients who had their first anorectal abscess revealed that more than half of them developed either recurrent abscesses or index perianal fistulas, with most events occurring during the initial follow-up period. The strongest and most consistent predictor of recurrence was abscess complexity, while preoperative imaging was also associated with increased risk. The low rate of intraoperative seton placement despite documented fistulas highlights variability in operative decision-making. These results demonstrate that early follow-up and standardized operative documentation and risk-based post-discharge planning are crucial for enhancing the results in this common surgical condition.

## AUTHOR’S CONTRIBUTION

SB was responsible for the study’s design, data collection and analysis, manuscript drafting, and approval of the final submitted version.

## CONFLICT OF INTEREST

The author has no conflict of interest to declare.

## Figures and Tables

**Table 1.** Baseline demographic and clinical characteristics of the study population (*n* = 302).

**Variable**	**Value**
**Age (years)**	37.9 ± 13.5
**Age group (years)**	
<40	184 (60.9%)
≥40	118 (39.1%)
**Gender**	
Male	229 (75.8%)
Female	73 (24.2%)
**BMI (kg/m^2^)**	27.6 ± 6.6
**BMI group (kg/m^2^)**	
<25	127 (42.1%)
≥25	175 (57.9%)
**Smoker**	14 (4.6%)
**Diabetes mellitus**	84 (27.8%)
**CRP (mg/dL)**	9.5 ± 9.3 (*n* = 222)
**CRP group**	
Normal (≤0.1 mg/dL)	8 (3.6%)
Abnormal (>0.1 mg/dL)	214 (96.4%)
**WBC(×10^9^/L)**	12.6 ± 5.9 (*n* = 299)
**WBC group**	
Normal (4–11 ×10^9^/L)	133 (44.5%)
Abnormal (<4 or >11 ×10^9^/L)	166 (55.5%)
**HbA1c (%)**	9.95 ± 2.77 (*n* = 81)
**HbA1c group**	
<6.5%	8 (9.9%)
≥6.5%	73 (90.1%)
**Follow-up duration (weeks)**	30.8 ± 44.5
**Time to abscess recurrence or fistula (weeks)**	19.5 ± 29.8 (*n* = 156)

Data are presented as mean ± SD (standard deviation) for continuous variables and n (%) for categorical variables.

**Table 2.** Operative and management characteristics of abscesses and fistulas (*n* = 302).

**Variable**	**Category**	**n (%)**
Abscess site	Perianal	249 (82.5%)
	Ischiorectal	43 (14.2%)
	Intersphincteric	6 (2.0%)
	Supralevator	3 (1.0%)
Abscess complexity	Simple	217 (71.9%)
	Complex	85 (28.1%)
Number of abscesses	Single	279 (92.4%)
	Multiple	23 (7.6%)
Intraoperative fistula detection	Detected	54 (17.9%)
	Not detected	248 (82.1%)
Seton placement	Yes	8 (2.6%)
Preoperative imaging	None	261 (86.4%)
	Ultrasound	2 (0.7%)
	CT	33 (10.9%)
	MRI	6 (2.0%)
Preoperative antibiotics	Yes	302 (100.0%)
Postoperative antibiotics	No	23 (7.6%)
	Yes	279 (92.4%)
Postoperative antibiotic duration (*n* = 276)	<7 days	186 (67.4%)
	7–14 days	76 (27.5%)
	>14 days	14 (5.1%)
Level of the surgeon	Resident	12 (4.0%)
	Specialist	159 (52.6%)
	Consultant	131 (43.4%)

**Table 3.** Chi-square analysis—predictors of abscess recurrence or fistula (binary outcome).

	**Recurrence/fistula *n* (%)**		
**Predictor variable**	**Yes**	**No**	*p*-value
**Age group (years)**			**0.033**
<40	86/184 (46.7%)	98/184 (53.3%)	
≥40)	70/118 (59.3%)	48/118 (40.7%)	
**Gender**			0.938
Male	118/229 (51.5%)	111/229 (48.5.0%)	
Female	38/73 (52.1)	35/73 (47.9%)	
**BMI group (kg/m^2^)**			0.401
<25	62/127 (48.8%)	65/127 (51.2%)	
≥25	94/175 (53.7)	81/175 (46.3%)	
**Smoking**			**0.020**
Yes	3/14 (21.4%)	11/14 (78.6%)	
No	153/288 (53.1%)	135/288 (46.9%)	
**Diabetes**			0.259
Yes	39/84 (46.4%)	45/84 (53.6%)	
No	117/218 (53.7%)	101/218 (46.3%)	
**CRP group (mg/dL)**			0.473
≤5	44/95 (40.4%)	51/95 (53.7%)	
>5	65/127 (51.2%)	62/127 (48.8%)	
**WBC group (×10^9^/L)**			0.345
≤11	64/133 (48.1%)	69/133 (51.9%)	
>11	89/166 (53.6%)	77/166 (46.4%)	
**HbA1c group (%)**			0.094
<6.5	6/8 (75%)	2/8 (25.0%)	
≥6.5	32/73 (43.8%)	41/73 (56.2%)	
**Abscess site**			0.194
Perianal	122/249 (49%)	127/249 (51.0%)	
Ischiorectal	26/43 (60.5%)	17/38 (39.5%)	
Intersphincteric	5/6 (83.3%)	1/6 (16.7%)	
Supralevator	2/3 (66.6%)	1/3 (33.3%)	
**Abscess complexity**			**<0.001**
Simple	92/217 (42.4%)	125/217 (57.6%)	
Complex	64/85 (75.3%)	21/85 (24.7%)	
**Number of abscesses**			0.358
Single	142/279 (50.9%)	137/279 (49.1%)	
Multiple	14/23 (60.9%)	9/23 (39.1%)	
**Level of the surgeon**			0.353
Resident	8/12 (66.7%)	4/12 (33.3%)	
Specialist	77/159 (48.4%)	82/159 (51.6%)	
Consultant	71/131 (54.2%)	60/131 (45.8%)	
**Preoperative Imaging**			**0.022**
Yes	28/41 (68.3%)	13/41 (31.7%)
No	128/261 (49%)	133/261 (51.0%)	
**Postoperative antibiotic**			0.452
No antibiotic	14/26 (53.8%)	12/26 (46.2%)	
<7 days	92/186 (49.5%)	94/186 (50.5%)	
7–14 days	40/76 (52.6%)	36/76 (47.4%)	
>14 days	10/14 (71.4%)	4/14 (28.6%)	

Bold values indicate statistical significance at *p* < 0.05.

**Table 4.** Binary logistic regression analysis—predictors of abscess recurrence or fistula.

**Predictor**	**OR**	**95% CI**	***p*-value**
Age	1.021	0.995–1.048	0.116
Gender	0.564	0.272–1.169	0.123
BMI	1.023	0.976–1.071	0.341
Diabetes	0.484	0.232–1.008	0.052
WBC	1.067	0.999–1.140	0.052
CRP	0.950	0.908–0.994	**0.028**
Preoperative imaging	2.987	1.208–7.383	**0.018**
Postoperative antibiotic duration	1.049	0.981–1.122	0.163

Bold values indicate statistical significance at *p* < 0.05.

**Table 5.** Cox proportional hazards regression model for predictors of time to abscess recurrence or fistula formation.

**Predictor variable**	**Univariable hazard ratio (95% CI)**	***p*-value**	**Multivariable hazard ratio (95% CI)**	***p*-value**
Age	1.005 (0.994–1.017)	0.360	1.000 (0.986–1.014)	0.978
Gender	1.004 (0.696–1.450)	0.982	0.954 (0.655–1.388)	0.806
Smoking	0.548 (0.175–1.721)	0.303	0.564 (0.177–1.798)	0.333
Diabetes	0.940 (0.654–1.352)	0.739	0.836 (0.557–1.257)	0.390
Abscess complexity	**2.260 (1.640–3.110)**	**<0.001**	**2.391 (1.658–3.450)**	**<0.001**
Preoperative imaging	1.470 (0.980–2.220)	0.065	0.936 (0.588–1.489)	0.780

Bold values indicate statistical significance at *p* < 0.05.
